# Degradation of Green Polyethylene by *Pleurotus ostreatus*

**DOI:** 10.1371/journal.pone.0126047

**Published:** 2015-06-15

**Authors:** José Maria Rodrigues da Luz, Sirlaine Albino Paes, Karla Veloso Gonçalves Ribeiro, Igor Rodrigues Mendes, Maria Catarina Megumi Kasuya

**Affiliations:** Departamento de Microbiologia, Universidade Federal de Viçosa, Viçosa, Minas Gerais, Brazil; Nanyang Technological University, SINGAPORE

## Abstract

We studied the biodegradation of green polyethylene (GP) by *Pleurotus ostreatus*. The GP was developed from renewable raw materials to help to reduce the emissions of greenhouse gases. However, little information regarding the biodegradation of GP discarded in the environment is available. *P*. *ostreatus* is a lignocellulolytic fungus that has been used in bioremediation processes for agroindustrial residues, pollutants, and recalcitrant compounds. Recently, we showed the potential of this fungus to degrade oxo-biodegradable polyethylene. GP plastic bags were exposed to sunlight for up to 120 days to induce the initial photodegradation of the polymers. After this period, no cracks, pits, or new functional groups in the structure of GP were observed. Fragments of these bags were used as the substrate for the growth of *P*. *ostreatus*. After 30 d of incubation, physical and chemical alterations in the structure of GP were observed. We conclude that the exposure of GP to sunlight and its subsequent incubation in the presence of *P*. *ostreatus* can decrease the half-life of GP and facilitate the mineralization of these polymers.

## Introduction

Due to the high *per capita* consumption, the accumulation of plastic waste in the environment is a primary source of pollution [1,2]. Plastic polymers are stable and barely undergo degradation in the cycle of nutrients of the biosphere [3]. It is estimated that polyethylene requires more than 100 years to be mineralized in the soil [4]. An alternative to reduce the accumulation of plastic wastes in the environment is the development of biodegradable plastic polymers that are economically viable and environment friendly.

In da Luz et al. [5], we showed that *Pleurotus ostreatus* can degrade and produce mushrooms using oxo-biodegradable plastic waste without any prior physical treatment. In da Luz [6], we evaluated the abiotic and biotic degradation of oxo-biodegradable plastic bags throughout 120 days of sunlight exposure and 90 days of fungal growth. Oxo-biodegradable polymers contain pro-oxidants and pro-degrading compounds [2,7] that are incorporated into the polymer chain to accelerate photo- or thermo-oxidation [2,7,8]. In this study, we investigated the degradation of green polyethylene by *P*. *ostreatus*. Green plastics are polymers developed from renewable raw materials such as sugarcane. Because plants capture and sequester carbon dioxide (CO_2_) during their growth, these biopolymers can help to reduce the emission of greenhouse gases [9]. These plastics can also be recycled [9]. However, there is no information available regarding the half-life or the degradation rate of these green polymers when discarded in the environment.

*P*. *ostreatus* is a lignocellulolytic fungus that can use lignin, cellulose and hemicellulose as carbon and energy sources [10–12]. This fungus has been used in the degradation of agroindustrial residues [10,13,14], the bioremediation of pollutants [12,15] and pulp bleaching [15]. The ability of lignocellulolytic fungi to degrade a large range of compounds is related to the high efficiency of their enzymatic system [14].

## Materials and Methods

Plastic bags were kindly donated by Fundação Arthur Bernandes. According to the manufacturer, these plastic bags are produced using low-density polyethylene and contain more than 50% green polymers that were developed from renewable raw materials. The information provided by the manufacturer regarding the types of polymers was confirmed by Fourier transform infrared spectroscopy (FTIR).

*Pleurotus ostreatus* PLO6 (GenBank accession number KC782771) used in this study belongs to the collection of the Department of Microbiology of the Federal University of Viçosa, MG, Brazil. The stock cultures were maintained on potato dextrose agar (PDA, Merck, Darmstadt, Germany). Mycelium of each stock culture were grown at 25°C on PDA agar in Petri dishes. After 15 d, mycelial disks were punched out with a 7 mm diameter cork borer and used to inoculate the substrates.

### 2.1 Abiotic degradation

The green polyethylene was exposed to sunlight for 30, 60, 90, and 120 days during the summer at a site protected from rainwater [6]. In this season, the duration of sunlight is 9 hours with a maximum intensity between noon and 3 hours. After each period of exposure, the plastic bags were submitted to physical and chemical analysis (see item 2.4).

### 2.2 Biotic degradation

Plastic bags were cut into fragments (5 cm x 1 cm), and 10 g of this material was placed in a 100 mL glass flask with 0.1 g of a commercially available paper towel [5]. Five milliliters of mineral medium [8,16] supplemented with 0.1 mL of filter-sterilized thiamine-HCl were added to the flasks. Four discs of agar containing the mycelium of *P*. *ostreatus* were inoculated into flasks, and the flasks were incubated at 25°C for 30, 60 or 90 days.

Plastic bags that had not been exposed to sunlight served as an experimental control.

### 2.3 Fungal respiratory activity and dry mass

The respiratory activity was measured by coupling the flasks to a continuous-flow respirometer coupled to an infrared CO_2_ detector (TR-RM8 Respirometer Multiplexer–Sable systems). CO_2_ measurements were performed every 24 h [17], and the flasks remained coupled to the respirometer throughout the study period.

To determine the dry mass, the flasks containing the fungal mycelium and substrates were dried at 105°C until a constant weight was obtained [6].

### 2.4 Analysis of the biodegradation of green polymers

The mineral composition of the green polyethylene before and after 120 days of exposure to sunlight was determined by SEM coupled with X-ray diffraction [5]. This technique is a semi-quantitative method and the percentage was calculated with base in the minerals analyzed (Table 1).

**Table 1:** Chemical composition of the green polyethylene before and after 120 d of exposure to sunlight.

Mineral Composition*	Exposure time to sunlight (days)	
	0	120
Si	41.19 A	35.83 B
Mn	8.51 A	7.41A
Fe	7.74 A	6.73 A
Co	6.31 A	5.49 A
Cu	3.05 A	2.65 A
Zn	8.08 A	7.03 A
Cd	22.06 A	19.19 B
N	2.80 A	2.44 A

* Relative concentration of elements ((weight %) at the surface of the plastic bags, analyzed by scanning electron microscopy coupled to X ray diffraction (semi-quantitative method). Means followed by different letters within the same line differ at Tukey test (P<0.05).

Physical changes, such as the formation of pits and cracks, and fungal colonization of the plastic surface were analyzed by SEM (Leo, 1430VP) with a magnification of 5000 X [5,7,18].

Alterations of the mechanical properties of the plastic were analyzed using universal testing equipment (Instron model 3367).

Chemical changes, such as the disappearance or formation of new functional groups and bond scission, were analyzed by FTIR [2,8,19]. The plastic strip fragment was placed in an appropriate support for solid sampling in the spectrophotometer (Thermo Nicolet).

### 2.5 Statistical analyses

The experiment followed a completely randomized design with five replicates. The data were subjected to analysis of variance (ANOVA). For chemical composition, mean values were compared by Tukey’s test, and dry mass and respiratory activity were subjected to regression analysis. These statistical analyses were performed using Saeg software (version 9.1, Universidade Federal de Viçosa) at a 5% significance value (p < 0.05). For mechanical properties, the average and standard deviation are presented.

## Results

### 3.1 Abiotic degradation

**Fig 1:**
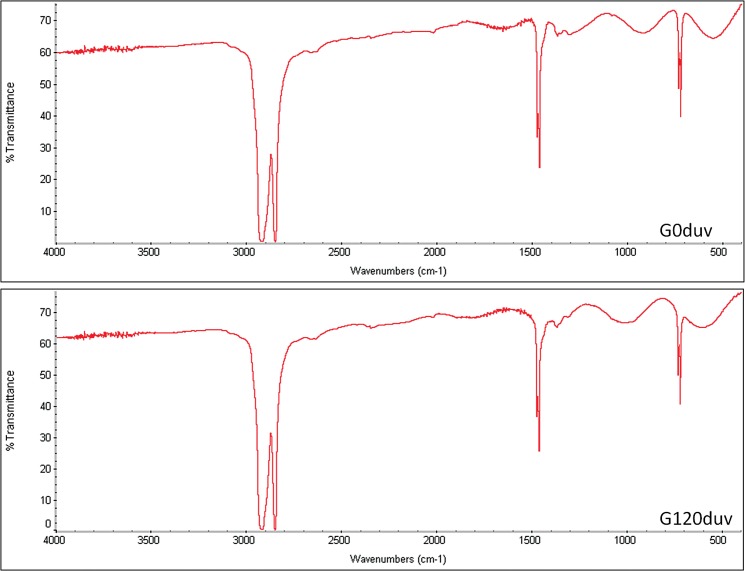
FTIR spectrum of the green polyethylene before (G0duv) and after 120 d of exposure to sunlight (G120duv). The bands: 3000–2500 cm^-1^ –Bond carbon-hydrogen (CH_2_ or CH_3_); 1500–1400 cm^-1^—Bond carbon-hydrogen (CH) and 800–700 cm^-1^—Bond carbon-carbon (C-C).

The bands of the FTIR spectrum before exposure to sunlight confirmed the information provided by the manufacturer that the plastic bags were produced using low-density polyethylene (Fig 1).

Silicon and cadmium were the main minerals found in the plastic bags used in this study (Table 1). Silicon can be involved in polyethylene polymerization by metallocene catalysts [20]. Some chemical elements that are essential for microbial growth, such as nitrogen, manganese, iron and zinc, were also detected (Table 1). Dyes that are commonly used in the production of commercial plastic bags may be the source of these and of the other elements shown in Table 1.

**Fig 2:**
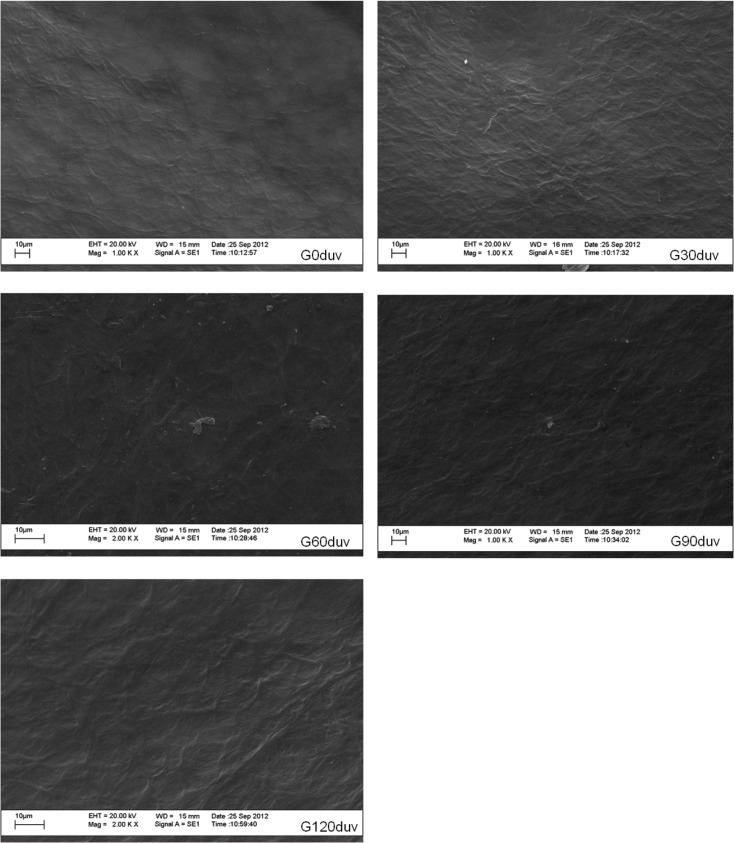
Scanning electron micrography of green polyethylene surface before (G0duv) and after 30 d (G30duv), 60 d (G60duv), 90 d (G90duv) or 120 d (G120duv) of exposure to sunlight. Note the absence of any expressive modification in the plastic surface due to sunlight exposure.

After 120 d of exposure to sunlight, we observed a significant decrease (p < 0.05) in the relative concentrations of silicon and cadmium (Table 1). No cracks, pits or new functional groups were observed after this period (Figs 1 and 2), demonstrating that sunlight was not able to induce the abiotic degradation of these green plastics. However, sunlight exposure altered the mechanical properties of the material (Table 2). After 90 d of exposure to sunlight, the plastic was much more fragile and brittle, and these characteristics prevented the determination of its mechanical properties (Table 2). This suggests that the exposure of GP to sunlight can promote some sort of modification, and this in turn may improve the biological degradation of these plastic polymers.

**Table 2:** Mechanical properties of the green polyethylene before and after 30, 60, 90 and 120 days of exposure to sunlight.

Mechanical properties	Exposure time to sunlight (days)			
	0	30	60	<90*
Maximum load of break (N)	3.119 ± 0.461	2.938 ± 0.347	2.718 ± 0.486	nd
Energy at break (J)	0.720 ± 0.010	0.490 ± 0.08	0.350 ± 0.02	
Tensile extension at break (cm)	5.193 ± 0.708	4.430 ± 0.654	4.198 ± 0.858	
Load at tensile strength (N)	2.067 ± 0.334	2.037 ± 0.761	1.743 ± 0.304	
Elastic modulus (MPa)	23.992 ± 2.567	20.905 ± 1.344	15.798 ± 0.861	

* nd- values were not determined due to the high fragility of the material.

### 3.2 Biotic degradation

**Fig 3:**
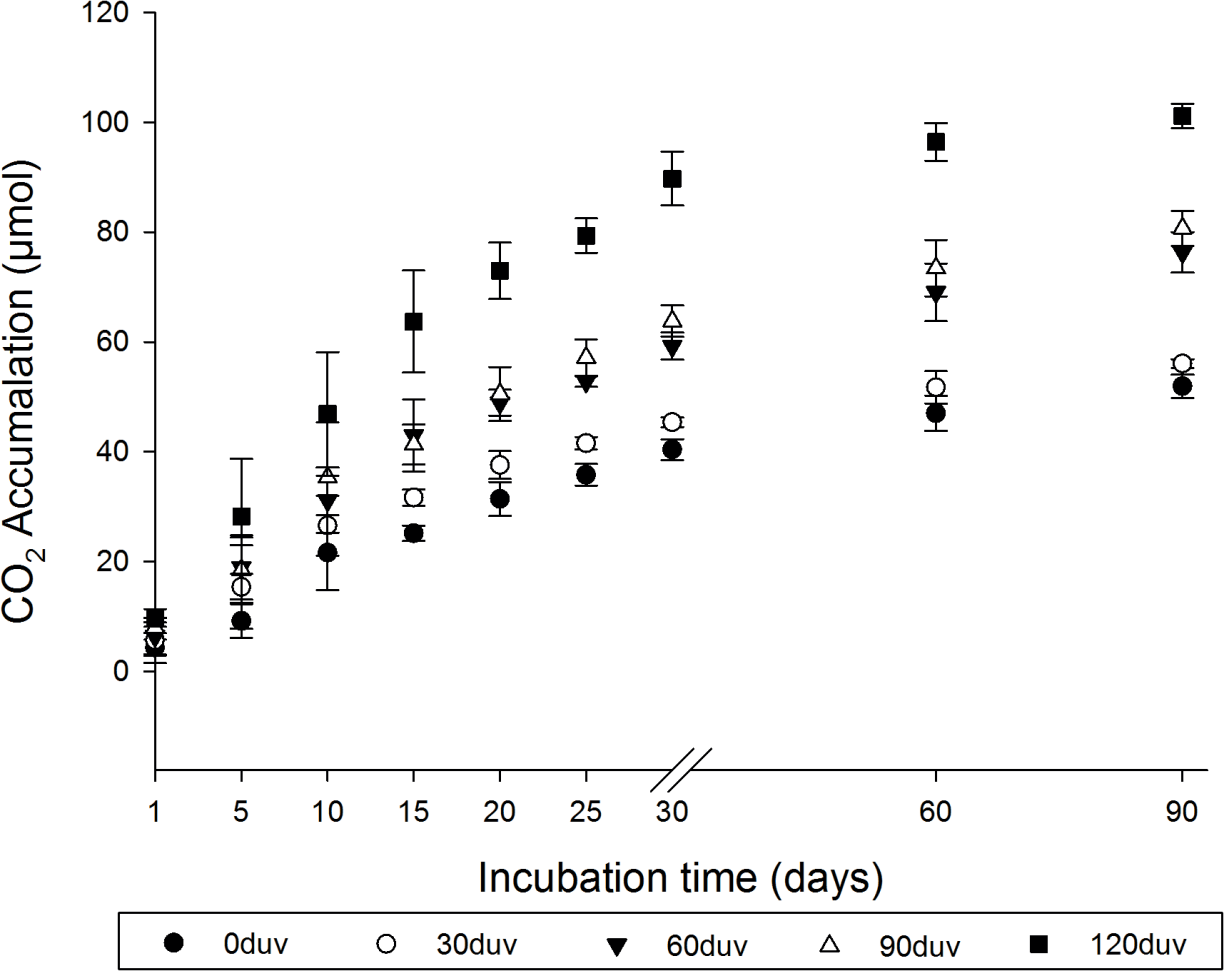
Respiratory activity of *Pleurotus ostreatus* grown during 90 days in green polyethylene before (Oduv) and after 30 d (30duv), 60 d (60duv), 90 d (90duv) or 120 d (120duv) days of exposure to sunlight.

The CO_2_ emissions from the microcosms inoculated with *P*. *ostreatus* were the highest in plastics with 120 d of exposure to sunlight (Fig 3). No differences (p > 0.05) in the CO_2_ emissions were observed between the microcosms with plastics exposed to sunlight for zero and 30 days, or for 60 and 90 days (Fig 3). These results shows that (a) *P*. *ostreatus* can grow independent of exposure to sunlight using green polyethylene as a substrate and (b) alterations of the mechanical properties caused by sunlight (Table 2) may have contributed to the fungal growth by increasing the contact surface.

The fungi caused the greatest reductions in the mechanical properties of plastics that had been previously exposed to sunlight for a longer period of time (Table 3). It was not possible to determine the mechanical properties (Table 3) or chemical alterations by FTIR due to the fragility of some of the plastic fragments.

**Table 3:** Mechanical properties of green polyethylene exposure or not to sunlight after 30, 60 or 90 d of incubation with *Pleurotus ostreatus*.

Mechanical properties	Time of incubation (days)	Exposition time of the ultraviolet light (days)		
		0	30	60
Maximum load of break (N)	30	2.839 ± 0.483	2.069 ± 0.311	0.548 ± 0.087
	60	1.728 ± 0.341	1.309 ± 0.305	nd
	90	nd		
Energy at break (J)	30	0.628 ± 0.058	0.420 ± 0.020	0.122 ± 0.031
	60	0.479 ± 0.043	0.401 ± 0.027	nd
	90	nd		
Tensile extension at break (cm)	30	5.021 ± 0.738	3.180 ± 0.952	1.390 ± 0.199
	60	4.425 ± 0.679	3.022 ± 0.843	nd
	90	nd		
Load at tensile strength (N)	30	1.761 ± 0.644	1.128 ± 0.313	0.258 ± 0.050
	60	1.352 ± 0.873	0.639 ± 0.150	nd
	90	nd		
Elastic modulus (MPa)	30	21.962 ± 4.771	15.798 ± 1.195	4.222 ± 0.781
	60	20.470 ± 3.341	10.073 ± 2.352	nd
	90	nd		

nd-values were not determined due to the high fragility of the plastic wastes.

**Fig 4:**
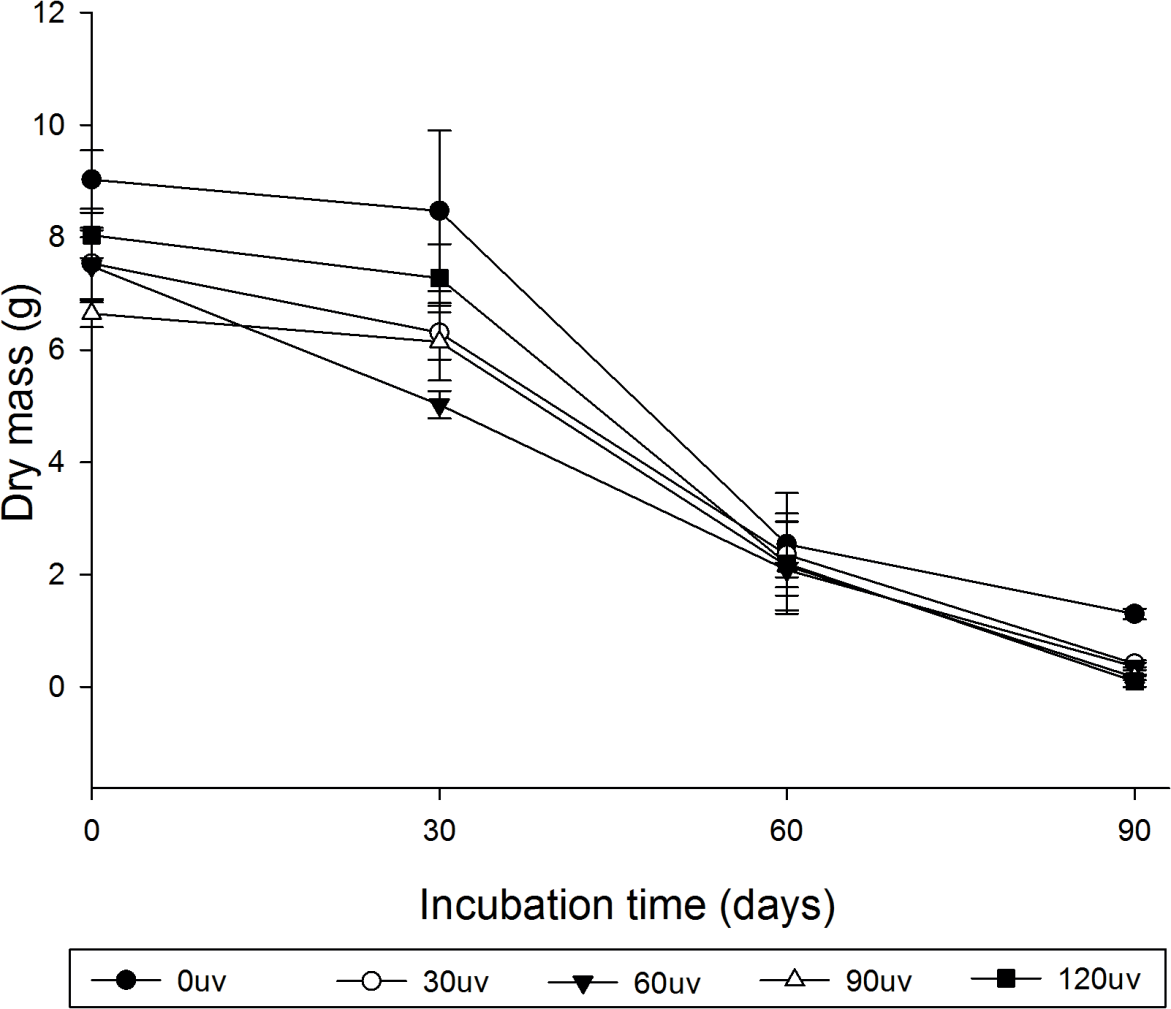
Dry mass of the substrate used by *Pleurotus ostreatus* for up to 90 d of incubation. Green polyethylene before (0duv) and after 30 d (30duv), 60 d (60duv), 90 d (90duv) or 120 d (120duv) of exposure to sunlight.

We observed a significant reduction in the dry mass of the substrates during the incubation period (Fig 4). This occurred independently of the period of exposure to sunlight (Fig 4). These results show that *P*. *ostreatus* could metabolize the green polyethylene without prior physical treatment.

**Fig 5:**
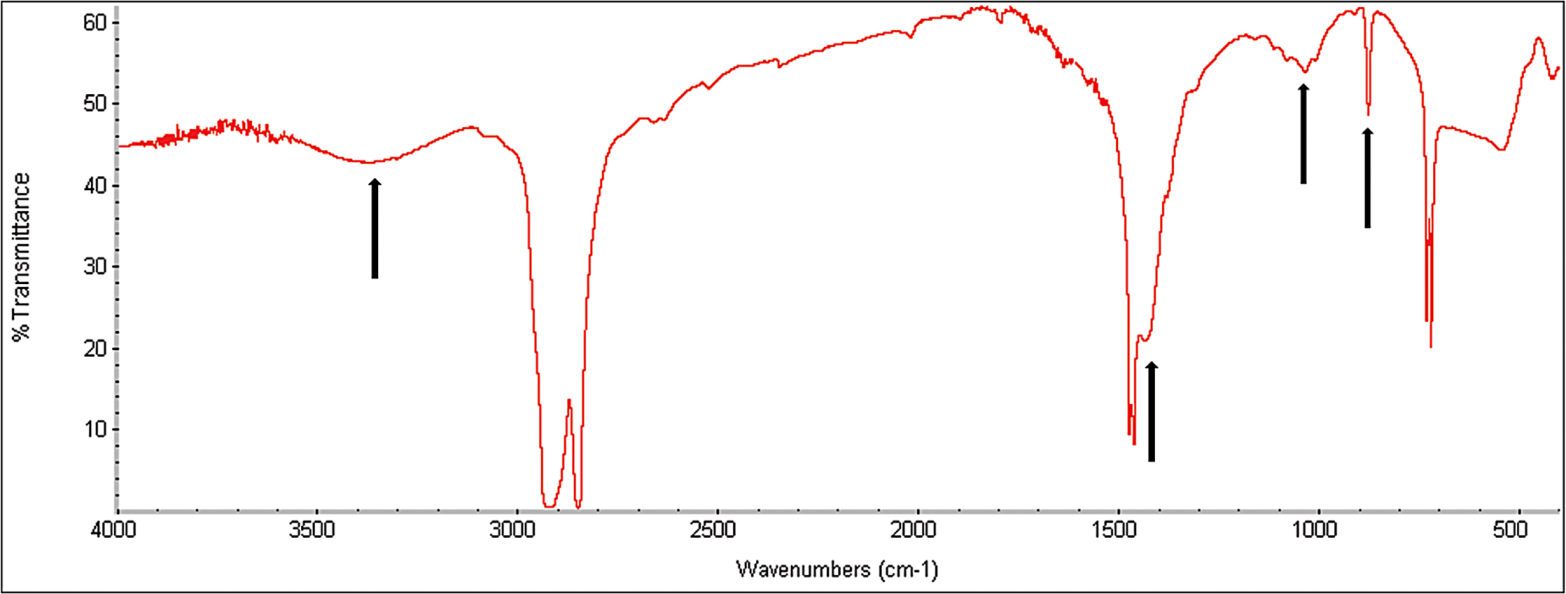
Espectrum of Fourier transform infrared spectroscopy of the green polyethylene after 120 d of exposure to sunlight and 90 d of incubation with *Pleurotus ostreatus*. The arrows show the bands that were observed after the fungal incubation (See also Fig 1).

The primary chemical alterations in the structure of the green polyethylene after fungal incubation resulted in the formation of four bands with wavelengths between 3500–3200 cm^-1^ and 1500–950 cm^-1^ (Fig 5). The band at 3500–3200 cm^-1^ is typical of a hydrogen-oxygen bond (OH), and the other bands are related to carbon-oxygen (CO), carbon-hydrogen (CH or CH_2_), and hydrogen-oxygen (OH) bonds.

**Fig 6:**
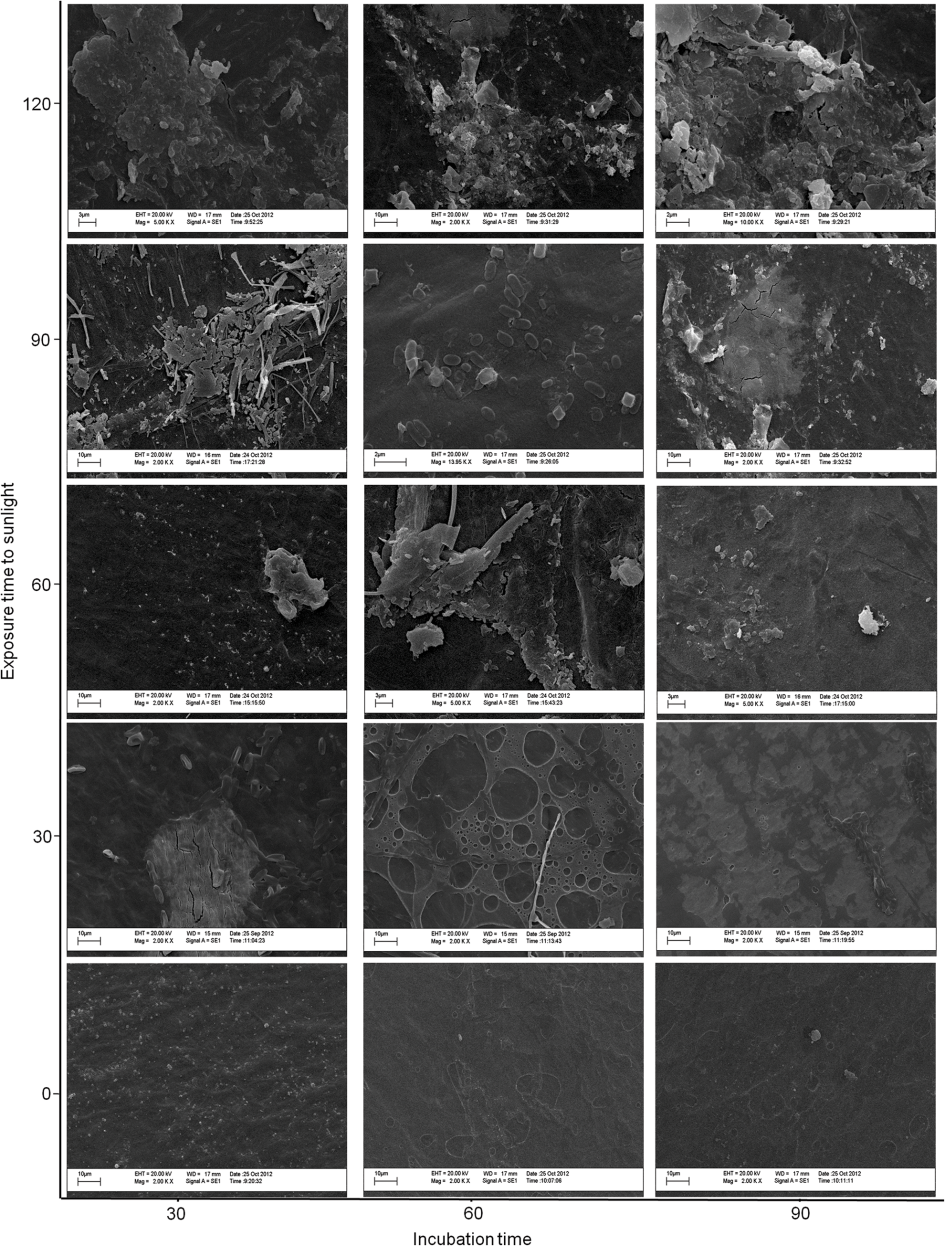
Scanning electron microscopy of green polyethylene exposed to sunlight for 0–120 d and incubated with *Pleurotus ostreatus* by 30, 60 or 90 d.

Regardless of exposure to sunlight, we observed physical alterations at the surface of the green polyethylene (Fig 6). However, these alterations were more evident in the polymers that were exposed to sunlight for a longer period before incubation with *P*. *ostreatus* than in the polymers without any previous sunlight exposure (Fig 6). These alterations confirm that green polyethylene can be degraded by *P*. *ostreatus*.

## Discussion

It has been suggested for at least 30 years that exposure to UV light, thermal heating or acid hydrolysis can initiate the thermal or photo-oxidation of polyethylene and thus promote fragmentation and possible microbial attack [2,18]. In this study, we did not observe any fragmentation or formation of new functional groups (Fig 1 and Fig 2) in the structure of green polyethylene after exposure to sunlight for up to 120 days. However, alterations in the mechanical properties of these polymers caused by sunlight (Table 2) were important for fungal growth (Fig 3) and for the biotic degradation of the green polyethylene (Fig 6). Thus, GP exposure to sunlight before composting or dumping in landfills may accelerate its biodegradation.

The degradation of a polymer in nature depends on various factors, such as humidity, light intensity, pH, the chemical structure of the polymer and the potential of microorganisms to use the polymer as a carbon or energy source [7,21,22]. In this study we showed the capability of *P*. *ostreatus* PLO6 to degrade green polyethylene (Fig 6). This potential was attributed to the complex of lignocellulolytic enzymes produced by this fungus. Several studies have suggested the participation of this enzymatic complex in the degradation of plastic polymers [23,24]. Da Luz et al. [5] showed that laccase, cellulase and xylanase are active during the growth of *P*. *ostreatus* on substrates containing oxo-biodegradable plastics and paper towels. According to these authors, the degradation of oxo-biodegradable polymers was primarily due to the activity of the laccases. Therefore, once conditions for microbial growth are established, principally those required for the growth of lignocellulolytic fungi, green polyethylene could be readily metabolized.

Nitrogen availability is a limiting factor for microbial growth [16,25]. The abundance of this element in the green polyethylene was lower than 3% (Table 1). Da Luz et al. [6] reported the presence of endomycotic nitrogen-fixing bacteria in the fungal hyphae of *P*. *ostreatus* PLO6. Other authors have also reported the presence of these bacteria in the hyphae of this species [26,27]. Thus, the growth of *P*. *ostreatus* (Fig 3) on green polyethylene may have been facilitated by the presence of these microorganisms that may have provided nitrogen for its growth. Yara et al. [27] suggested that interactions between nitrogen-fixing microorganisms and *P*. *ostreatus* can be an important factor in bioleaching and bioremediation.

Da Luz et al. [6] suggested that the degradation of oxo-biodegradable plastics by *P*. *ostreatus* was possibly due to (a) the presence of pro-oxidant ions in the polymers, (b) the low specificity of the lignocellulolytic enzymes for their substrate and (c) the presence of endomycotic nitrogen-fixing microorganisms in the *P*. *ostreatus* fungal hyphae. In our study, 50% of the green polymers were derived from sugarcane and served a substitute for the pro-oxidant ion. The low specificity of the lignocellulolytic enzymes could be observed in the degradation of green polyethylene because green polyethylene has little to no structural similarity with lignocellulosic compounds. The low specificity of these enzymes has also been shown to allow for the biodegradation of many pollutants, including some recalcitrant molecules [28–31]. Roldán-Carrillo et al. [32] also showed the degradation of plastic polymers containing starch by enzymes produced by *Phanerochaete chrysosporium*.

The chemical alterations observed in this study have also been shown in others studies [5,19,33]. These alterations are evidence of the oxidation of green polyethylene by *P*. *ostreatus*.

Physical alterations on the surface of green polyethylene (Fig 6) and the loss of dry mass (Fig 4) are additional evidence for the mineralization of the substrate by *P*. *ostreatus*.

## Conclusions

Regardless of exposure to sunlight, *Pleurotus ostreatus* PLO6 can degrade green polyethylene. The exposure of the plastic to sunlight and its subsequent incubation in the presence of the fungus could be an alternative way to decrease the half-life of plastic waste in dumps or landfills.
